# Examination of the Teeth

**Published:** 1853-07

**Authors:** 


### *Examination of the Teeth.*

Dentists are frequently called upon to examine a set of teeth for the purpose of ascertaining whether they require any operation, such as filling, scaling, &c., and it is to be regretted that such requests are not made oftener by our patients, as too many of them take special care to keep away from their dentists as long as possible, and only make their appearance, slowly, and reluctantly, after being reminded of their duty by a painful twinge, a "grumbling," or an uneasy sensation in one or more of their teeth, which is generally the indication of an exposed nerve. These requests generally come at

**Aug.* 28.—To-day I saw Mrs. J. She informed me that the right, superior central molar, gave her considerable pain at one time during July, since which time it has not been painful, although not as comfortable as before this period. On examining the tooth I am satisfied that the nerve is destroyed. This is the last tooth in Mrs. J's. mouth operated on, all of which have failed.

an inconvenient moment, when the dentist is engaged with his patient in the chair, and every minute of time is taken up. Under such circumstances, there is no doubt but what the proper course would be to insist upon the patient taking an appointment, coming again, and submitting to a thorough examination; but if the dentist proposes this, he is at once met with the question: "Can't you just look at them? Why, it won't take you a minute." Under the force of this appeal he too often submits: gives them a hasty examination and pronounces them in good condition, and the patient departs much better pleased than if the answer had been that half a dozen needed filling, which is very probably the case.

Now, we wish to caution all our readers against these hasty examinations of the teeth, as the consequence too often is loss of business, loss of patients, and loss of reputation. Loss of business, by frequently getting only the fee for extracting the tooth, soon after, which might have been filled at the time of the hasty and careless examination. Loss of patients by sacrificing that confidence which they repose in their dental advisers, and loss of reputation by the story getting abroad, accompanied by the accusation of gross carelessness, loss of eye sight, or too much business, and growing rich and independent.

A lady once called upon us and requested that her teeth might be examined, without saying that she had just come from the office of another dentist (which was the case) who had told her that they were all in good order and needed no operation. We happened to be disengaged at the time, gave them a thorough examination, and found four teeth that required filling, two (lower bicuspids) decayed to the pulp and in an aching condition. Another came in with a small quantity of salivary calculus burrowing under the edge of the gums around the inferior incisors, which were beginning to loosen. Her teeth had also been recently pronounced in good condition. We removed the tartar, and to this day we have the credit, whether justly or not, of having saved her teeth Still another recently told us that she employed Dr. ——, until he pronounced a tooth sound that another dentist extracted the next day, to relieve her from excruciating pain. Let it be borne in mind that these cases occurred with women, some of whom have *tongues* as well as teeth, and to our certain knowledge, the stories have been often repeated; but these cases are too frequent to require further specification. All have, undoubtedly, either suffered or been benefitted in this way, in proportion to their care or carelessness.

Too much care cannot be bestowed when examining teeth for the purpose of discovering caries, as when situated upon the approximal surfaces of the teeth, it is often very difficult to discover until the decay has made such progress as to endanger the loss of the tooth. This is, perhaps, most frequently the case with the lower bicuspids and molars. The approximal surfaces of these teeth are slightly convex, both longitudinally and transversely, and generally have their point of contact near the middle, it is near this point that caries commence, and as the surfaces around it are so close together, it is often difficult to pass the smallest point into the cavity. In such cases the decay may generally be detected by passing the floss silk between them, and if the enamel be decomposed the rough edge will cut the silk. In the incisors, caries may generally be discovered by the discoloration which it produces, and the same appearance will sometimes be seen in those molars and bicuspids which are of a translucent and pearly hue. If proper care be observed during the examination, caries may always be detected, either by the appearance of the teeth, with the fine pointed probe, or the floss silk, before it has made so much progress as to endanger the tooth.

Discoloration of the enamel is not always decay. It is often produced upon the anterior approximal surfaces of the first molars, by the decay of the second molars of the temporary set, and in a few cases will remain through life without the enamel becoming decomposed. When this is the case, the second teeth are of a firm, dense structure, and not pre-disposed to decay; but in most teeth this discoloration, if in contact with the bicuspis, end in decay, and if the teeth be delicate, it will be better practice to remove it with the file, taking care to so file them that the surface cannot come in contact with the tooth from which it has been separated.

When teeth have been once separated and filled upon their approximal surfaces, they are apt to close again, unless a shoulder is left on each tooth near the gum. Owing to the shape of the teeth in some cases, or the extent of the caries, it is not always practicable to leave such a shoulder, and the teeth will come together again, and caries recommence at the point of contact. When called to examine teeth thus situated, we should always find the edge of the filling nearest the gum, with our probe, otherwise we can never be sure that decay is not going on at this point. Both dentist and patients have heretofore been so much afraid of the free use of the file, and so little judgment and skill have been displayed in separating teeth, preparatory to filling, that we frequently find them filled and crowded close together. In all such cases we should make it a point to separate them again, in such manner that we can see and feel with the probe all around the fillings, the teeth can then be pronounced sound and in good condition.

In the early period of our practice we were once called to examine a set of teeth to see if any operation was needed. They had been well filled by one of the best dentists in the country. Among the fillings was one on the approximal surface of a superior molar, and towards the masticating surface of the tooth the filling was solid, well finished, and the dentine healthy around it. It was also separated at this part, from the surface of the adjoining tooth, but the upper part of the filling, and the portion of the tooth above it was so close to the other, that a probe could not be passed between them. Deceived by the perfect condition of that part of the filling that could be seen, and not having learned from experience the danger of leaving it in this situation, we pronounced it to be in good condition. Judge of our surprise and mortification, when a few weeks after, the patient called again, with the gold filling forced up into a large cavity above, the nerve exposed, and the tooth so exceedingly painful that it was necessary to extract it.

This case serves to illustrate hundreds that we have since seen, and which, like it, might not have been discovered until caries had penetrated to the pulp, if we had not separated them again, as far as the filling extended, so as to thoroughly examine every part of it.

Examinations of the teeth should be made at least semi-annually, from the cutting of the first molar to the age of twenty-five, or as long as the teeth show signs of active decay, and time should be taken to make them thorough and methodical, beginning with one side of the lower jaw, proceeding from tooth to tooth around the mouth, and returning by the upper. When it is completed the dentist should be sure, from positive knowledge, before he pronounces the teeth in good condition. So far as we have known, it is not customary to charge a fee for examination of the teeth, unless it is found necessary to perform some operation. How far this may influence the dentist, and induce careless and hasty examinations, we will not say; but in our opinion there is no good reason why we should not be remunerated for time and skill spent in this way, and we think that if more time and care were generally bestowed upon them, that no liberal person would expect to receive them gratuitously.

[The above very sensible remarks we take from the Recorder, and would urge the importance of the subject. We have recently been astonished at several cases, where teeth had been condemned by dentists of some repute; which, on close and careful inspection, we found in safe condition for filling; two or three cases of this kind had remained unplugged for a year, after having been condemned. The teeth had been somewhat troublesome—painful when foreign matter was forced upon the carious portion—cold water produced pain, and the so oft repeated expression was taken for granted, "the nerve is exposed." The teeth had been probed and their nerves pronounced exposed.

In matters of this kind we take nothing for granted, because our patient thinks so; almost every day we see the fallacy of this. We regard it as no evidence of an exposed nerve, if the tooth has been painful. If, however, there has been continued tooth-ache, loss of sleep, and the tooth becomes sore, we generally suppose the nerve is exposed, or that the disease has reached the nerve, and inflammation of this has supervened. If the tooth has become too long (as the expression is), the peri-dental membrane is generally involved in the disease. We believe with the editor of the Recorder, that plenty of time should be taken, and that we should charge for the service. Time is money, and advice is often far better than any operation we can give.—Ed.]

